# Neuropathic pain after spinal intradural benign tumor surgery: an underestimated complication?

**DOI:** 10.1007/s10143-022-01775-7

**Published:** 2022-03-28

**Authors:** Vicki Marie Butenschoen, Annika Nehiba, Bernhard Meyer, Maria Wostrack

**Affiliations:** Department of Neurosurgery, School of Medicine, Klinikum Rechts Der Isar, Technical University Munich, Ismaningerstr. 22, 81675 Munich, Germany

**Keywords:** **N**europathic pain, Spine surgery, Intradural tumor

## Abstract

Neuropathic pain presents a burdening and impairing condition which may occasionally occur after spinal tumor surgery. While it has been described in peripheral nerve sheath tumors, data on other intradural tumor patients is sparse. We hereby present a large cohort population undergoing different intradural spinal tumor surgery with assessment of early postoperative and follow-up outcomes, focusing on the occurrence of neuropathic pain. We performed a retrospective monocentric study including all patients treated for intradural spinal tumors between 2009 and 2020. We extracted surgical aspects as well as pre- and postoperative clinical courses from the records. Statistical analysis of potential contributing prognostic factors was performed including matched pair analysis. In total, 360 patients were included for analysis. At a median follow-up of 2 years, 26/360 patients complained of a neuropathic pain syndrome (7.2%) requiring continuous medication. Of these patients only 50% complained preoperatively of pain. Tumor entity did not significantly influence the incidence of postoperative neuropathic pain (*p* = 0.91). Sacrifice of the tumor carrying nerve root and tumor recurrence also did not increase the risk for this condition. Persistent neuropathic pain requiring continuous treatment occurred in 7.2% of patients undergoing intradural spinal surgery in our cohort. This frequently underestimated postoperative adverse event represents a disabling condition leading to a substantial impairment in the quality of life among the affected patients.

## Introduction

Postoperative neuropathic pain syndromes, defined as “pain initiated or caused by a primary lesion or dysfunction in the nervous system”[21] following a GABAergic pathway[12], may significantly affect the quality of life of patients suffering from lesions occupying spinal space such as arachnoid cysts or tumors as well as after spinal cord injury [2, 13, 23] [8–10, 16]. The rate of the occurrence of postoperative neuropathic pain in terms of painful radiculopathy ranges between 20 and 40%, depending on the type and location of the surgical approach, and remains difficult to treat [20] [4, 8, 14]. In intradural tumor surgery, particularly in nerve sheath tumors, the loss of Schwann cell guidance leading to a random sprouting of neurons was assumed as a possible underlying cause for the development of neuropathic pain [20, 22]. Occurrence of neuropathic pain after other intradural tumor surgery is still the subject of scattered case reports [10].

While most published literature focuses on overall and progression-free survival and postoperative functionality [5], long-term disabilities such as postoperative neuropathic pain in tumor patients in need of the administration of pain medication such as neuroleptics are often underestimated. Our aim was to study the burden of postoperative neuropathic pain in patients suffering from various spinal intradural neoplasms and to describe and assess potential prognostic factors increasing the probability of its occurrence.

## Methods

### Study population

We performed a monocentric retrospective cohort study of all patients undergoing surgical treatment for spinal intradural tumors between April 2009 and July 2020. Patients harboring malignant or high-graded tumors such as anaplastic ependymomas WHO grade III, astrocytomas WHO grade II and higher-graded, or intradural metastases were excluded from the analysis due to their aggressive behavior with a distinctly more complex pain syndrome.

First, we assessed preoperative data such as gender, age, type and duration of symptoms, and comorbidities. Secondly, we retrieved intraoperative information such as duration of surgery, surgical approach, and complications from the medical records. Postoperative follow-up assessment included the presence of postsurgical pain syndromes focusing on the occurrence of neuropathic pain with the need of pharmaceutical treatment, particularly including specific medication targeting neuropathic pain such as *Gabapentine* or *Carbamazepine,* as well as the neurological and clinical status using the Karnofsky Performance Status Scale (KPS). Tumor entities including tumor grading according to the World Health Organization (WHO) System were captured. We analyzed the extent of resection (EOR), location of the tumor regarding the spinal cord (intradural intramedullary vs. extramedullary), surgical information (root amputation, midline vs. lateral incision of the spinal cord), and the spinal level (cervical vs. thoracic vs. lumbar spine). The approach was classified into unilateral approach (laminotomy, hemilaminectomy, foraminotomy), laminectomy, laminoplasty, and dorsal fixation. We assessed the postoperative course and extracted long-term data from the follow-up examinations.

### Statistics

Statistical analyses were performed using SPSS Statistics 26 (IBM, Chicago, IL). We compared categorical data using the chi-square test or Fisher’s exact test as needed. Mean values were compared using the independent samples *t* test. To identify prognostic risk factors for neuropathic pain, we performed a matched pair analysis including all patients with a burdening neuropathic pain syndrome and matching patients without postoperative pain symptoms with corresponding age and tumor localization.

The association between potential factors and the occurrence or persistence of preexisting neuropathic pain (last follow-up data or discharge data for those with missing follow-ups) was analyzed using ANOVA and linear regression modeling. We assumed the following factors to be potentially predictive: complete nerve root amputation, age, intra- vs. extramedullary tumors, preoperative radicular pain, gender, incomplete vs. gross total resection (GTR), and symptom duration. To assess the correlation, we used Kendall’s Tau correlation coefficient. All tests were performed two-sided, and a p-value < 0.05 was considered significant.

### Ethical considerations

We executed the presented study in accordance with the ethical standards outlined in the Declaration of Helsinki. Also, we obtained a positive vote by a local ethics committee beforehand (number 5766/13). Due to the retrospective nature of the study, prospective patient consent was not required and waived by our local ethics committee.

## Results

### Patient population

In total, 486 surgeries were performed for benign intradural tumor resection between April 2009 and July 2020 in our neurosurgical department. Complete follow-up data was available on 385/486 (79.2%), and after reviewing the records, 10 patients resulting in 25 cases were additionally excluded because of previous tumor surgery on the same spinal level and duplicates or triplicates. In total 360 patients were included for further analysis.

Overall, 197/360 patients were female (54.7%), and 163/360 patients were male (45.3%). Median age was 53 years (IQ range 40–66 years).

The most common entities were benign peripheral nerve sheath tumors (124/360, 34.4%), ependymomas WHO° I-II (92/360, 25.6%), and spinal meningioma (91/360, 25.3%). Other tumor entities included intramedullary hemangioblastoma WHO grade I (22/360, 6.1%), pilocytic astrocytoma WHO°I (9/360, 2.5%), and paraganglioma (22/360, 6.1%) (Table 1).

**Table 1:** Patient tumor entities operated upon, most patients suffered from peripheral nerve sheath tumors (34.4%) followed by ependymoma (25.6%) and meningioma (25.3%)

	Frequency (*n*)	Percent (%)
Meningioma	91	25.3
Peripheral nerve sheath tumor	124	34.4
Ependymoma	92	25.6
Hemangioblastoma	22	6.1
Astrocytoma	9	2.5
Others	22	6.1
Total	360	100

Preoperative pain symptoms were present in 67.1% of the patients at the initial presentation in the outpatient department. Preoperative motor deficits were present in 27.2%, sensory deficits in 39.4%, and vegetative deficits in 11.6% of the patients.

### Surgical data

A unilateral approach (including laminotomy, hemilaminectomy, foraminotomy and costotransversectomy) was performed on 252/360 patients (70%). In 42/360 patients, a laminectomy was performed (11.7%), and in 53/360 patients (14.7%), we opted for a laminoplasty to achieve tumor resection. In 12/360 patients, we performed a retroperitoneal (10/12) or transthoracic (2/12) approach. In addition to the posterior approach, one patient required a dorsal fixation (Table 2).

**Table 2:** Surgical approaches performed for tumor resection, with 70% of the patients undergoing a unilateral approach (laminotomy, foraminotomy, or hemilaminectomy)

	Frequency (n)	Percent (%)	Cumulative percent
Unilateral	252	70	70
Laminectomy	42	11.7	81.7
Laminoplasty	53	14.7	96.4
Stabilisation	1	0.3	96,7
Transthoracic/retroperitoneal	12	3.3	100
Total	360	100	

Mean surgery duration was 157 min (range 54–499 min). Overall, 326/360 required only one operation to achieve tumor resection (90.6%), and 34/360 patients underwent 2 or more surgeries for complete tumor resection (9.4%). Most surgeries were performed at the thoracic (137/360, 38.1%) or lumbar level (113/360, 31.4%), followed by the cervical spine (70/360, 19.4%), the thoracolumbar junction (16/360, 4.4%), sacral tumors (14/360, 3.9%), and the cervicothoracic junction (10/360, 2.8%). In 34.7%, the dura was sutured with a 5.0 Gore-Tex suture in a watertight manner; in 65.3% of the patients, the suture was covered by an absorbable fibrin sealant (TachoSil).

Median length of hospital stay was 6.5 days (range 1–43 days, IQ 5–10 days). Intraoperative neuromonitoring was performed in 95 patients (26.4%).

### Outcome and neuropathic pain

The median preoperative and postoperative Karnofsky performance index (KPS) was 90%. GTR could be achieved in 312/360 (86.7%) of cases. Tumor recurrence was observed in 19/360 cases (5.3%), ranging from 0% in hemangioblastoma patients to 4.8% in peripheral nerve sheath tumor and 6.5% in ependymoma patients.

In our cohort study, 26/360 patients suffered from neuropathic pain requiring a medication at follow-up (7.2%), describing tingling and burning sensations as well as paresthesia either in one extremity or a specific dermatome.

Neuropathic pain was mostly observed in patients undergoing a combined dorsal/retroperitoneal tumor resection (16.7% vs. 6.9%, *p* = 0.212, OR 2.7) and patients undergoing surgery at the cervical or thoracic level (8.6% and 9.5%, *p* = 0.090) without reaching statistical significance. The occurrence of neuropathic pain was not associated with tumor recurrence (*p* = 0.493) or the extent of resection (GTR *p* = 0.764). Although patients suffering from neuropathic postoperative pain were predominantly female (17/26 patients, 65.4%), the difference was not significant (*p* = 0.257). Depending on tumor entity, the percentage of patients suffering from postoperative neuropathic pain ranged from 0 (pilocytic astrocytoma) to 7.3% (peripheral nerve sheet tumor) and 8.8% (meningioma) (Table 3, *p* = 0.91). While patients with entirely extramedullary tumors also suffered from neuropathic pain, the amount was higher in patients suffering from intramedullary tumors, but the difference did not reach statistical significance in the whole cohort analysis (12.5% vs. 7.3%, *p* = 0.097) (Table 3). The number of segments addressed was similar among patients suffering from neuropathic pain and patients without postoperative pain syndrome (mean 1.82 vs. 1.77 segments, *p* = 0.801). Interestingly, patients suffering from postoperative neuropathic pain had neurological deficits in 73.1% of the cases (57.6% suffering from sensory deficits, 30.7% motor deficits), and the amount was lower in patients without neuropathic pain (58.1%, *p* = 0.097, 37.3% sensory deficits, 27% motor deficits).

**Table 3:** Risk factors for the occurrence of postoperative neuropathic pain with corresponding OR (odds ratio) and *p* values (without reaching significance)

Risk factor	% patients with pain	OR	*P* value
Approach retroperitoneal	16.7	2.70	0.212
Meningioma	8.8	1.33	0.489
Tumor location cervical or thoracic	9.2	2.10	0.104
Preoperative pain	4.8	0.53	0.130
GTR	7.1	0.83	0.764
Intramedullary tumor	12.5	2.14	0.097
Female sex	8.6	1.62	0.309

In subgroup analysis, patients suffering from ependymoma had a higher risk for postoperative neuropathic pain at the cervico-thoracic location (14.3% vs. 0%, *p* = 0.01) and if tumors were intramedullary (16.2% vs. 12.5% in intra/extramedullary vs. 0% in extramedullary ependymoma, *p* = 0.005). The EOR and number of segments affected was not associated with the occurrence of neuropathic pain. In patients suffering from spinal meningioma, the location of the tumor again affected the occurrence of postoperative neuropathic pain (cervico-thoracic 10.3% pain vs. 0%) and incomplete tumor resection was associated with a higher risk of postoperative neuropathic pain without reaching statistical significance (GTR 7,2% vs. 25%, *p* = 0.09). Again, the number of segments did not affect postoperative neuropathic pain.

Neuropathic pain did not resolve spontaneously in any of the affected patients during a median follow-up of 24 months (range 2–10 years).

In matched pair analysis, neuropathic pain significantly influenced the clinical status. Median KPS at follow-up was 100% in patients without neuropathic pain compared to a median KPS of 90% in patients suffering from neuropathic pain (*p* = 0.007). Detailed assessment of all patients describing a burdening neuropathic pain revealed no association of the pain with spinal nerve root amputation or any other of the applied potential risk factors in multivariate analysis including the surgical approach, tumor location, sex or extent of resection (*p* = 0.602, *p* = 0.562, *p* = 0.773 and *p* = 0.685).

## Discussion

Only a few studies address the incidence of neuropathic pain after spinal tumor resection [4, 8], while it presents a mostly burdening complication after intradural spinal tumor surgery and was observed in 7.2% of the patients postoperatively, with the highest occurrence rate in patients undergoing surgical resection of peripheral nerve sheath tumors, meningioma but also in spinal ependymoma patients. (Table 4).

**Table 4:** Occurrence of postoperative neuropathic pain depending on tumor entity, with highest amount of pain encountered in meningioma (8.8%) and ependymoma patients (7.6%)

		Meningioma	Peripheral Nerve tumor	Ependymoma	Hemangioblastoma	Astrocytoma	others	Total
Pain	Count	8	9	7	1	0	1	26
	% within Pain	30,8%	34,6%	26,9%	3,8%	0%	3,8%	100%
	% within Histology	***8,8%***	***7,3%***	***7,6%***	***4,5%***	***0%***	***4,5%***	***7,2%***
	% of Total	2,2%	2,5%	1,9%	0,3%	0%	0,3%	7,2%

Although published literature discusses the occurrence of neuropathic pain mainly in peripheral nerve sheath tumors [4], we also identified this complication in other patients, undergoing resection of spinal meningioma and ependymoma. Compared to other studies, the rate of postoperative neuropathic pain in our cohort population ranged between 5 and 10%, depending on tumor entity, and was therefore much lower than previously described in other publications stating that almost one third of the patients undergoing intramedullary ependymoma surgery suffer from postoperative neuropathic pain [17]. The lower rate described in our study may be biased due to the retrospective nature of our data, relying on written reports with a possible underreporting of neuropathic pain in follow-up documentation, mainly focusing on tumor recurrence and neurological deficits. Furthermore, neuropathic pain lacks a precise definition [3, 9]. In our study, we only included patients requiring pain medication which may underestimate the actual burden of disease. Patients with paresthesia without regular administration of analgesics, and patients suffering from back pain were therefore not classified as patients suffering from neuropathic pain.

While literature mostly focuses on root pain resolving and the improvement of symptoms after tumor resection [11], in our patient cohort, only 14/26 patients also described a preoperative radicular pain. In 12/26 patients the pain described at follow-up occurred as a new symptom only after surgical tumor resection (48.1%).

We could not identify any significant potential risk factors leading to a higher risk of postoperative neuropathic pain among our patients, while female sex, thoracic level, and intramedullary tumors seem to be more at risk, without reaching statistical significance. The lack of statistical significance may be caused by the small number of patients suffering from neuropathic pain at follow-up, but we did not identify any risk factor in matched pair analysis. Possible triggering factors suggested by other authors may be intraoperative hypotension and perioperative administration of corticoids [16]. Unfortunately, we did not address these factors retrospectively.

### Treatment of neuropathic pain

In our cohort population, patients describing burdening neuropathic pain were administered medication such as anti-epileptic drugs *Pregabalin, Carbamazepine* or *Gabapentin*. Two patients were additionally given an antidepressant (*Amitriptyline*). Occasionally, in cases of acute exacerbation of the pain, the patient was advised to receive a periradicular corticosteroid injection. Additional promising treatment options for neuropathic pain which have been described recently are spinal cord stimulation by an implanted pulse generator or repetitive transcranial magnetic stimulation [1, 3].

Unfortunately, literature on postoperative neuropathic pain is sparse. While most studies aim to describe the clinical status in terms of muscle strength, sensation, and functional independence, the postoperative disability caused by impairing pain is often underrepresented [25]. All our patients described a significant burden and reduced quality of life with the necessity of a regular pain medication. Conservative treatment such as physiotherapy and manual therapy were tried but unsuccessful. Pain remained stable during the follow-up years and was first described shortly after surgery, similar to other published results [9].

## Limitations

We only included patients with follow-up in our neurosurgical department, which may distort the rate of complications and pain syndrome due to a possible selection bias. Furthermore, our study was performed retrospectively, therefore not investigating whether there is a real causative relationship between the occurrence of neuropathic pain and the performed surgery.

Neuropathic pain may occur in patients suffering from preoperative diabetes mellitus [19], peripheral arterial occlusive disease, or cancer-related pain [7, 24]. In our cohort, we did not specifically analyze the comorbidities of our patients, possibly biasing the results described and presenting a strong limitation of our study.

Unfortunately, we did not perform any drug response analysis, nor did we assess the medication dosage. Due to the retrospective nature of the study, we cannot provide data on patient variability. Furthermore, neuropathic pain was diagnosed if the patient reported on a radicular burning and/or tingling painful sensation. Due to the retrospective nature of the study, the patients did not answer a specific neuropathic pain questionnaire, nor did we assess the timing of onset of the pain. The pain remained present in all patients during follow-up, requiring long-term pain medication. The intractable nature of neuropathic pain has been described in numerous studies [6], describing modest results of pharmacotherapy with high risks of medication side effects. Unfortunately, we did not investigate the potential benefit of neuromodulative interventions in cases of persistent neuropathic pain, a therapeutic option gaining more and more importance in current literature[18].

Of course, including all patients suffering from benign spinal intradural tumors creates a strong heterogeneity regarding the acquired data. While pathological mechanisms of the occurrence of neuropathic cannot be drawn from our studied population, our aim was to report on its occurrence in other spinal tumors as most case reports and case series only include peripheral nerve sheath tumors and ependymomas. The heterogeneity presents a limitation, but our goal was to remain as broad as possible to include all spinal pathologies and to describe the prevalence of neuropathic pain, including also subgroup analysis.

Neuropathic pain has been associated with reduced ability to work and a markedly impaired quality of life [15]. Unfortunately, we did not assess and quantify the disability encountered using specific questionnaires and only rely on written medical reports for follow-up examinations.

In our study, we did not investigate the influence of postoperative neuropathic pain on reintegration to work or other daily living activities, but we would assume a significant negative impact on the quality of life among the affected patients.

## Conclusion

In total, 7.2% of patients complained of postoperative neuropathic pain at follow-up examinations and required regular pain medication at follow-up. Neuropathic pain occurred after various intradural spinal surgeries and should therefore be mentioned as a possible complication preoperatively. Patients were especially at risk when undergoing peripheral nerve sheath tumor, meningioma or ependymoma resection compared to other benign intradural spinal tumors.

## Data Availability

The datasets used and/or analyzed during the current study are available from the corresponding author on reasonable request.
